# Targeting the Sirtuin–1/PPAR–Gamma Axis, RAGE/HMGB1/NF-κB Signaling, and the Mitochondrial Functions by Canagliflozin Augments the Protective Effects of Levodopa/Carbidopa in Rotenone-Induced Parkinson’s Disease

**DOI:** 10.3390/medicina60101682

**Published:** 2024-10-14

**Authors:** Mennatallah A. Elkady, Ahmed M. Kabel, Lamees M. Dawood, Azza I. Helal, Hany M. Borg, Hanan Abdelmawgoud Atia, Nesreen M. Sabry, Nouran M. Moustafa, El-Shaimaa A. Arafa, Shuruq E. Alsufyani, Hany H. Arab

**Affiliations:** 1 Pharmacology Department, Faculty of Medicine, Tanta University, Tanta 31527, Egypt; mennah.elkady@med.tanta.edu.eg; 2 Medical Biochemistry Department, Faculty of Medicine, Tanta University, Tanta 31511, Egypt; lamees.dawood@med.tanta.edu.eg; 3 Department of Histology and Cell Biology, Faculty of Medicine, Kafrelsheikh University, Kafrelsheikh 33516, Egypt; aza_hlal2014@med.kfs.edu.eg; 4 Physiology Department, Faculty of Medicine, Kafrelsheikh University, Kafr El-Shaikh 33516, Egypt; hany_borg@med.kfs.edu.eg; 5 Department of Pharmacology and Toxicology, College of Pharmacy, University of Hail, Hail 2440, Saudi Arabia; h.soliman@uoh.edu.sa; 6 Department of Biochemistry and Molecular Biology, Faculty of Pharmacy, Al-Azhar University, Cairo 35527, Egypt; 7 Clinical Oncology Department, Faculty of Medicine, Tanta University, Tanta 31527, Egypt; nesreen.afefy@med.tanta.edu.eg; 8 Medical Microbiology & Immunology Department, Faculty of Medicine, Ain Shams University, Cairo 11566, Egypt; nouran.m@dau.edu.sa; 9 Basic Medical Science Department, College of Medicine, Dar Al Uloom University, Riyadh 13314, Saudi Arabia; 10 College of Pharmacy and Health Sciences, Ajman University, Ajman 346, United Arab Emirates; e.arafa@ajman.ac.ae; 11 Center of Medical and Bio-Allied Health Sciences Research, Ajman University, Ajman 346, United Arab Emirates; 12 Department of Pharmacology and Toxicology, College of Pharmacy, Taif University, P.O. Box 11099, Taif 21944, Saudi Arabia; s.alsofyani@tu.edu.sa (S.E.A.); h.arab@tu.edu.sa (H.H.A.)

**Keywords:** canagliflozin, levodopa/carbidopa, Parkinson’s disease, sirtuin-1/PPAR-gamma axis, pathophysiology, mice

## Abstract

*Background and Objectives:* Parkinson’s disease (PD) is a pathological state characterized by a combined set of abnormal movements including slow motion, resting tremors, profound stiffness of skeletal muscles, or obvious abnormalities in posture and gait, together with significant behavioral changes. Until now, no single therapeutic modality was able to provide a complete cure for PD. This work was a trial to assess the immunomodulatory effects of canagliflozin with or without levodopa/carbidopa on rotenone-induced parkinsonism in Balb/c mice. *Materials and Methods:* In a mouse model of PD, the effect of canagliflozin with or without levodopa/carbidopa was assessed at the behavioral, biochemical, and histopathological levels. *Results:* The combination of levodopa/carbidopa and canagliflozin significantly mitigated the changes induced by rotenone administration regarding the behavioral tests, striatal dopamine, antioxidant status, Nrf2 content, SIRT–1/PPAR–gamma axis, RAGE/HMGB1/NF-κB signaling, and mitochondrial dysfunction; abrogated the neuroinflammatory responses, and alleviated the histomorphologic changes induced by rotenone administration relative to the groups that received either levodopa/carbidopa or canagliflozin alone. *Conclusions:* Canagliflozin may represent a new adjuvant therapeutic agent that may add value to the combatting effects of levodopa/carbidopa against the pathological effects of PD.

## 1. Introduction

Parkinson’s disease (PD) is a pathological state characterized by a combined set of abnormal movements including slow motion, resting tremors, profound stiffness of skeletal muscles, or obvious abnormalities in posture and gait [1]. Among the most common neurodegenerative disorders worldwide, PD occupies second place [2]. The characteristic pathognomonic feature of PD is the massive death of the dopaminergic neurons in the substantia nigra of the brain [3]. Several mechanisms were thought to participate, to a great extent, in these pathologic events; including disturbed pro-oxidant/antioxidant state, induction of mitochondrial dysfunction, widespread neuroinflammation, and perturbation of autophagy/apoptosis balance in the brain tissues [4,5].

The recent literature describes a number of animal models that are commonly utilized in ongoing research in PD [6]. These models include rotenone-induced, 6-hydroxydopamine-induced, 1-methyl-4-phenyl-1,2,3,6-tetrahydropyridine-induced, and genetic models [7]. Rotenone is a naturally toxic substance that is used extensively in PD research, owing to its ability to induce dopaminergic neuron loss and reproduce changes that mimic many clinical features of PD in humans [8]. The toxic effects of rotenone on the substantia nigra were thought to originate from its inhibitory effects on the mitochondrial functions in the brain, leading to marked suppression of mitochondrial complex I activity and ATP formation [9]. In addition, rotenone-induced aggregation of α-synuclein in the nervous system was thought to induce massive neuroinflammation with subsequent appearance of the classical signs of PD such as bradykinesia, resting tremors, and rigidity [10]. Moreover, the abnormally increased levels of reactive oxygen species (ROS) detected in the substantia nigra of animals treated with rotenone suggests that disturbance of the redox state in the brain tissues is an additional mechanism by which rotenone may induce the classical features of PD [11].

Sirtuin-1 (SIRT1) is a member of the sirtuin family of proteins with a histone deacetylase activity that regulates many essential physiological functions in the human body [12]. The observation that SIRT1 levels are significantly decreased in PD patients directed the scope of the recent research towards the exploration of the possible effects of SIRT1 on the pathogenesis of PD [13]. The ability of SIRT1 to regulate proteins involved in autophagy, such as AMP-activated protein kinase and mammalian target of rapamycin (mTOR) may significantly mitigate the pathogenic events related to the suppression of autophagy in PD [14]. In addition, SIRT1 maintains the deacetylated state of peroxisome proliferator-activated receptor-γ coactivator-1α (PGC-1α) with subsequent inhibition of ROS production and protection of the neurological tissues from oxidative damage and mitochondrial dysfunction [15]. Furthermore, the deacetylating activity of SIRT1 on nuclear factor-kappa B (NF-κB) may significantly contribute to the potential amelioratory effects of SIRT1 on neuroinflammation, which is an important pathognomonic feature of PD [16].

High mobility group box 1 (HMGB1) is an intracellular chromatin-associated protein that is involved in a variety of physiological processes that play a role in cellular homeostasis [17]. Being released from the inflammatory cells, HMGB1 was proven to participate in the neuroinflammatory changes that are frequently encountered in PD; an action mediated via NF-κB transcription [18]. Furthermore, the ability of HMGB1 to affect the redox state and to modulate autophagy and apoptosis strengthens its role in the pathogenesis of PD [19]. Interestingly, the targeting of HMGB1 with different therapeutic agents had proven efficacy in mitigation of the neurodegenerative changes in animal models of PD [20]. 

Levodopa is the precursor of dopamine, which has been used for years as a classic therapeutic agent for the management of PD [21]. Once administered, levodopa passes the blood–brain barrier (BBB) and is converted by central dopa decarboxylase to dopamine, which enhances dopaminergic transmission in the brain neurons and stimulates the dopaminergic receptors in the striatum [22]. Due to the fact that only 5% of the administered levodopa can enter the brain, carbidopa is added to levodopa to counteract its peripheral metabolism and enhance its ability to cross the BBB, thereby decreasing the required doses and the anticipated adverse effects [23]. The beneficial effects of levodopa in PD are derived from its ability to restore dopamine levels in the brain and to counteract the deleterious effects of ROS and neuroinflammation together with its ability to modulate the apoptotic changes in the brain neurons [22,24]. However, the long-term use of levodopa may carry the risk of induction of a wide range of adverse effects and even tolerance [21].

Sodium-glucose cotransporter 2 (SGLT2) inhibitors have recently proven their efficacy in the management of hyperglycemia in patients with type 2 diabetes mellitus [25]. Among them, canagliflozin had exhibited promising properties for combatting many of the neurological disorders, independent of its antidiabetic effects [26]. These effects may rely on the ability of canagliflozin to attenuate neurotoxin-induced ROS production in addition to interference with the inflammatory signals that are responsible for the production and release of proinflammatory mediators in the brain tissues of patients with neurodegenerative disorders, including PD [27]. Unlike other SGLT2 inhibitors, canagliflozin has a unique ability to significantly affect MAP kinase signaling, which regulates the balance between apoptosis and autophagy in the brain tissues [26]. Taken together, these reports represent the keys to determine the objectives of the current study; these were to investigate the extent to which canagliflozin with or without levodopa/carbidopa can affect rotenone-induced PD in mice and to delineate the potential mechanistic pathways that may contribute to these effects.

## 2. Materials and Methods

### 2.1. Ethical Statement

All the experiments performed in the current study complied with the ARRIVE guidelines and were carried out in accordance with the U.K. Animals (Scientific Procedures) Act, 1986 and associated guidelines; and EU Directive 2010/63/EU for animal experiments. Furthermore, reporting of animal testing experiments complied with the ARRIVE guidelines. Ethical approval of this study was issued by the Research Ethics Committee of Tanta Faculty of Medicine, Tanta University, Egypt (Approval code 36264PR622/3/24).

### 2.2. Chemicals, Reagents, and Drugs

Rotenone was provided by Sigma Aldrich Co., St. Louis, MO, USA (CAS number 83-79-4). Canagliflozin and carbidopa were obtained from LGC Standards, Manchester, NH, USA (CAS numbers 842133-18-0 and 38821-49-7, respectively). Levodopa was purchased from Cayman Chemical, Ann Arbor, MI, USA (CAS number 59-92-7). Spectrum Chemical Mfg. Corp., New Brunswick, NJ, USA was the source of dimethyl sulfoxide and methyl cellulose (CAS numbers 67-68-5 and 9004-67-5, respectively). Rotenone was freshly prepared daily by being dissolved in 1% DMSO and then diluted in sunflower oil to reach a final concentration of 2.5 mg/mL. Canagliflozin and levodopa/carbidopa were suspended in 1% methyl cellulose solution.

### 2.3. Animals

Fifty Balb/c male mice obtained from the animal house of the Faculty of Science, Tanta University, Egypt (7 weeks old, body weight 22–28 g) were kept in wire mesh cages (temperature 23 ± 3 °C, humidity of 56 ± 6%, 12:12 light-dark cycle) for one week to acclimatize, with free access to food and water.

### 2.4. Rotenone-Induced Parkinson’s Disease Model

In the present work, all animals except the control ones were, daily, injected intraperitoneally with rotenone at a dose of 2.5 mg/kg for 4 weeks, to induce Parkinsonian-like symptoms, as described by Alabi et al. [28].

### 2.5. Grouping of Animals

**Figure 1:**
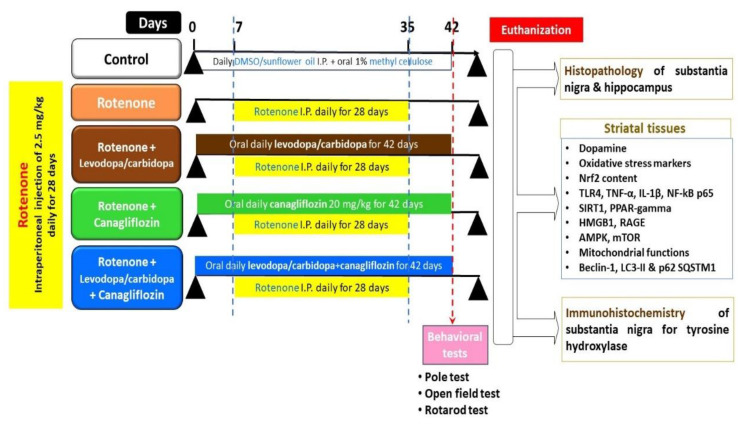
An illustrative representation of the experimental protocol of the study.

A research assistant who was blinded to the experimental protocol of the current study was asked to randomly divide the animals into five equal groups (10 mice per group) as follows: control animals were intraperitoneally injected with DMSO/sunflower oil once daily for 4 weeks and received daily oral 1% methyl cellulose solution by oral gavage starting 1 week before and continued for 5 weeks after initiation of DMSO/sunflower oil injection; rotenone group; rotenone group treated with oral daily L-dopa (10 mg/kg)/carbidopa (30 mg/kg) by oral gavage [29]; rotenone group treated with daily canagliflozin (20 mg/kg) by oral gavage [30]; and rotenone group treated with oral daily levodopa/carbidopa concomitantly with oral daily canagliflozin in the aforementioned doses by oral gavage. Treatment of animals with levodopa/carbidopa or canagliflozin was initiated 1 week before and continued for 5 weeks after the initiation of rotenone injections (Figure 1).

### 2.6. Determination of the Changes in Behavior of the Studied Groups

One week following the last rotenone injection, the animals were tested for behavioral changes using the pole test, open field test, and the rotarod test.

Bradykinesia, which is an important manifestation of PD, was assessed by the pole test. In three testing sessions, the animals were put on the top of a pole (diameter 1 cm, height 50 cm) and the time taken by the animal to climb down to the floor was determined, and then the average time of these three sessions was recorded, as described by Glajch et al. [31].

**Figure 2:**
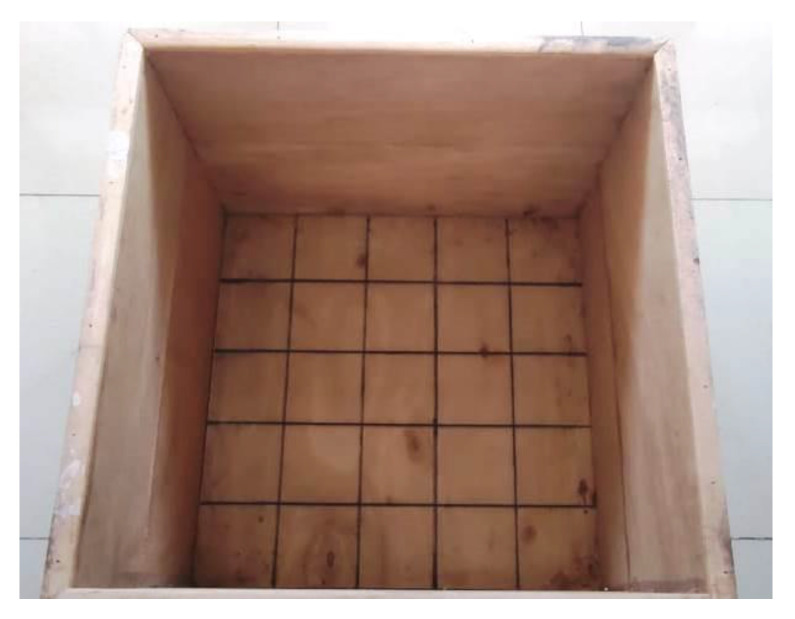
The open field test apparatus.

The ability to move spontaneously and exploratory behavior were tested using the open field test, in which a white plywood apparatus (dimensions 72 cm × 72 cm × 36 cm) with one wall and the floor made of clear Plexiglas, was used to allow tracking of animals’ movements (Figure 2). The floor of the apparatus was divided by lines into 25 squares, with a central square. The animals were allowed to acclimatize in the apparatus for 20 min before starting the test. Open field software obtained from Clever Sys Inc., Reston, VA, USA was used to track and record the overall distance traveled by each animal (as an indicator of the extent of motor performance), immobile latency (to assess bradykinesia), and rearing movements (to assess the exploratory behavior) for five minutes, as explained by Ishola et al. [32]. Grooming and stretching postures were used as indicators of anxiety behavior [33].

Motor coordination and balance were assessed using the rotarod test, in which a rotarod apparatus (Orchid Scientific & Innovative India Pvt Ltd., Ambad, Nashik, India) composed of thin steel rods (Diameter 3 cm) arranged in a cylindrical form rotate at a constant speed of 20 rpm. The animals were subjected to three training sessions before the test to enable them to maintain their posture on the rotarod. After that, the testing session was carried out, in which the rotarod started at 0 rpm, accelerated to 20 rpm within 1 minute, and then maintained a constant speed of 20 rpm for 4 min. The time taken by each animal to fall from the rotarod was recorded [34].

### 2.7. Specimens’ Collection and Processing

After performing the behavioral tests, all animals were euthanized by cervical dislocation and the brain was excised and temporarily frozen at −80 °C. The corpus striatum of each hemisphere was dissected and homogenized in 400 μL antioxidant solution, as described by Bentea et al. [35]. After that, the yielded homogenate was centrifuged for 15 min at 8000× *g* at 4 °C and the resulting supernatant was withdrawn and underwent further processing for quantification of the biochemical parameters in the striatal tissues.

### 2.8. Assessment of Dopamine Levels in the Striatal Tissues

The tissue levels of striatal dopamine were assessed using ELISA kits obtained from MyBioSource, Inc., San Diego, CA, USA (Catalogue number MBS269234) following the vendor’s instructions.

### 2.9. Determination of the Effect of Different Treatments on the Nuclear Factor (Erythroid-Derived 2)-Like 2 (Nrf2) Content and the Redox State of the Striatal Tissues

The striatal tissue content of Nrf2 was quantified using kits purchased from Elabscience, TX, USA (Catalogue number E-EL-M2607). Tissue reduced glutathione levels were assayed according to Nuhu et al. [36]. Tissue glutathione peroxidase (GPx) was determined in the striatal tissues using ELISA kits provided by LSBio, Shirley, MA, USA (Catalogue number LS-F11568). Kits provided by antibodies-online.com, Limerick, PA, USA (Catalogue number ABIN773241) were used for the assay of glutathione-S-transferase (GST) in the striatal tissues, according to the provider’s protocol.

### 2.10. Quantification of the Levels of Toll-like Receptor 4 (TLR4), Tumor Necrosis Factor Alpha (TNF-α), Interleukin 1 Beta (IL-1β), and Nuclear Factor Kappa B (NF-kB) p65 in the Striatal Tissues

The levels of striatal TLR4 and NF-kB p65 were determined using ELISA kits purchased from Elabscience, TX, USA (Catalogue numbers E-EL-M2417 and E-EL-M0838 respectively). Boster Biological Technology, Pleasanton, CA, USA was the source of sandwich ELISA kits used for quantification of the striatal tissue content of TNF-α and IL-1β (Catalogue numbers EK0527 and EK0394, respectively).

### 2.11. Assessment of the Striatal Tissue Levels of SIRT1 and Peroxisome Proliferator-Activated Receptor (PPAR)-Gamma in the Studied Groups

SIRT1 levels in the striatal tissue specimens were assessed using kits purchased from Biorbyt Ltd., Cambridge, CB4 0WY, UK (Catalogue number orb408848). LSBio, Shirley, MA, USA provided ELISA kits that were used for the determination of PPAR-gamma levels in the striatal tissues (Catalogue number LS-F4267).

### 2.12. Determination of the Striatal Tissue Levels of HMGB1 and Receptors of Advanced Glycation End Products (RAGE) in the Studied Groups

The striatal tissue levels of HMGB1 were determined using kits provided by ABclonal, Woburn, MA, USA (Catalogue number RK06737). Sandwich ELISA kits purchased from Bio-Techne, Minneapolis, MN, USA (Catalogue number MRG00) were used to assess the striatal tissue levels of RAGE.

### 2.13. Quantification of the Striatal Tissue Levels of Mitochondrial ATP, Mitochondrial Complex-I Activity, and Mitochondrial Transmembrane Potential in the Studied Groups

The method of differential centrifugation explained by Liao et al. [37] was utilized to perform proper isolation of the mitochondria of the striatal tissues. Mitochondrial ATP levels in the striatal tissues were quantified using kits purchased from MyBioSource, Inc., San Diego, CA, USA (Catalogue number MBS267352). These kits utilized the double antibody sandwich ELISA technique, in which the plate wells are precoated with an anti-mouse ATP monoclonal antibody, while the detection antibody is a biotinylated polyclonal antibody. The samples were added to the plate wells, followed by the biotinylated antibodies, and then washed out with PBS or TBS. After that, Avidin-peroxidase conjugates were added to the plate wells. After washing, TMB substrate was added to form a blue product which was converted to a yellow color after the addition of the stop solution. The intensity of the formed color and ATP concentration in the sample were positively correlated. Biorbyt Ltd., Cambridge, CB4 0WY, UK was the source of the ELISA kits used for assessment of the mitochondrial complex-I activity (Catalogue number orb1668354). The principle of assessment depends on the hypothesis that mitochondrial complex-I catalyzes the dehydrogenation of NADH to NAD^+^. Therefore, the rate of NADH oxidation measured at 340 nm was used to determine the mitochondrial complex-I activity. The mitochondrial transmembrane potential was determined according to the method described by Maity et al. [38]. Briefly, the isolated mitochondria were incubated with JC-1 (300 nM) in JC-1 assay buffer in the dark for ten minutes at 37 °C. Then, the fluorescence of each sample was determined in a RF-6000 spectrofluorometer, Shimadzu Corporation, USA (excitation, 490 nm; slit, 5 nm; emission, 590 nm for J-aggregate and 530 nm for J-monomer; slit, 7.2 nm).

### 2.14. Assessment of the Striatal Tissue Levels of AMPK and mTOR in the Studied Groups

MyBioSource, Inc., San Diego, CA, USA supplied the kits used for the assessment of AMPK levels in the striatal tissues (Catalogue number MBS2505028). The levels of mTOR in the striatal tissues were assayed using ELISA kits purchased from Abcam, Waltham, MA, USA (Catalogue number ab206311).

### 2.15. Determination of the Effect of the Different Treatments on the Autophagy State of the Striatal Tissues

Beclin-1 levels in the striatal tissues were quantified following the instructions enclosed with kits supplied by MyBioSource, Inc., San Diego, CA, USA (Catalogue number MBS724152). Cell Signaling Technology, Inc., Danvers, MA, USA was the provider of ELISA kits used for assessment of the autophagy marker LC3-II levels in the striatal tissues (Catalogue number 35172). Kits supplied by Novus Biologicals, LLC, Centennial, CO, USA (Catalogue number NBP2-61300) were the tools used for the determination of p62 SQSTM1 content of the striatal tissues.

### 2.16. Assessment of the Effect of Canagliflozin with or without Levodopa/Carbidopa on the Histopathological Changes of the Brain Tissues Induced by Rotenone Administration

The formalin-fixed hippocampus and substantia nigra specimens undergo dehydration in graded concentrations of ethyl alcohol. Then, they were cleared with xylol and fixed in paraffin wax. After that, the paraffin blocks were sliced by a rotatory microtome that yields slices of 5 µm thickness. These slices were deparaffinized by xylol then hydrated in descending grades of ethanol. Thereafter, these sections were stained with hematoxylin obtained from Bio-Techne, Minneapolis, MN, USA (Catalogue number 5222). After two minutes, these sections were washed in tap water and then stained with eosin purchased from Santa Cruz Biotechnology, Inc., Dallas, TX, USA (CAS number 17372-87-1) for 30 s. After that, these sections were visualized under a light microscope (KERN & SOHN, Albstadt, Germany) and the average thickness of the different areas of the hippocampus was quantified using ImageJ software (Version 1.52a, U.S. National Institute of Health, Bethesda, MD, USA).

### 2.17. Detection and Quantification of the Changes in Tyrosine Hydroxylase (TH) Immunoexpression in the Substantia Nigra

The paraffin-embedded sections from the substantia nigra were immersed in xylene to be deparaffinized, then rehydrated in descending grades of ethanol. After that, these sections were incubated in 10% hydrogen peroxide solution for 15 min in order to block the endogenous peroxidase activity. Then, they were incubated with a preheated citrate buffer solution for half an hour for antigen retrieval, after which they were incubated with monoclonal anti-TH primary antibodies purchased from Novus Biologicals, LLC, Centennial, CO, USA (1:200; Catalogue number MAB7566) overnight at 4 °C. After that, the nuclei were counterstained with hematoxylin and the slides were examined under a light microscope (KERN & SOHN, Germany, objective lens OBB-A1595, 100×) connected to a digital camera. TH immune expression was evaluated using ImageJ software purchased from Bethesda, MD, USA. The number of TH-positive neurons was counted in six non-overlapping fields from each slide and the mean value for each mouse was recorded.

### 2.18. Statistical Assessment of the Obtained Data

The statistical package for the social sciences (SPSS) version 21.0 was the statistical tool used for analysis of the results of the present study, which were expressed as mean ± standard deviation (S.D.). One-way analysis of variance (ANOVA) followed by Dunnett’s post hoc test were utilized to detect the presence of statistically significant differences between the studied groups. A *p*-value of 0.05 was taken as the critical point below which the obtained data denoted statistical significance.

## 3. Results

### 3.1. Effect of Canagliflozin with or without Levodopa/Carbidopa on the Behavioral Changes Induced by Rotenone Administration

**Figure 3:**
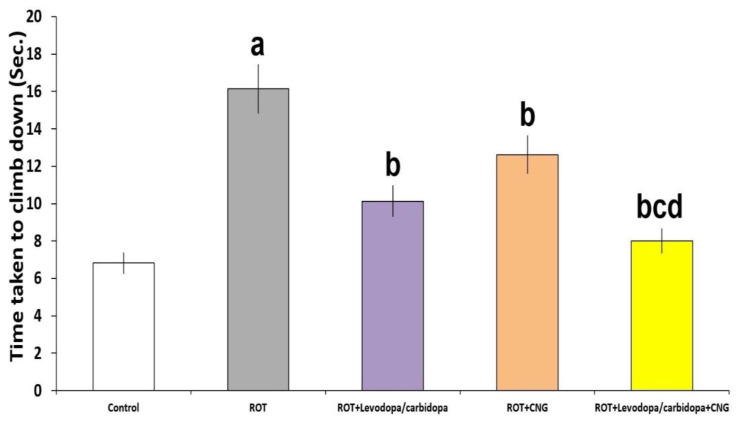
Effect of levodopa/carbidopa with or without canagliflozin on rotenone-induced changes in the pole test (Mean ± SD). ^a^ Significant compared to the control group (*p*-value less than 0.05); ^b^ significant relative to rotenone group (*p*-value less than 0.05); ^c^ significant relative to rotenone + levodopa/carbidopa group (*p*-value less than 0.05); ^d^ significant relative to rotenone + canagliflozin group (*p*-value less than 0.05). ROT (rotenone); CNG (canagliflozin).

As depicted in Figure 3, administration of rotenone elicited a significant prolongation of the time taken by the animal to climb down from the top of the pole to the floor relative to the control animals (*p* < 0.001). Administration of either levodopa/carbidopa or canagliflozin significantly shortened the time taken by the animal to climb down from the top of the pole to the floor when compared with mice treated with rotenone alone (*p* = 0.012 and *p* = 0.03, respectively). The group treated with levodopa/carbidopa concomitantly with canagliflozin showed significant shortening of the time taken by mice to climb down from the top of the pole to the floor, relative to mice treated with each of these agents alone (*p* < 0.01).

**Figure 4:**
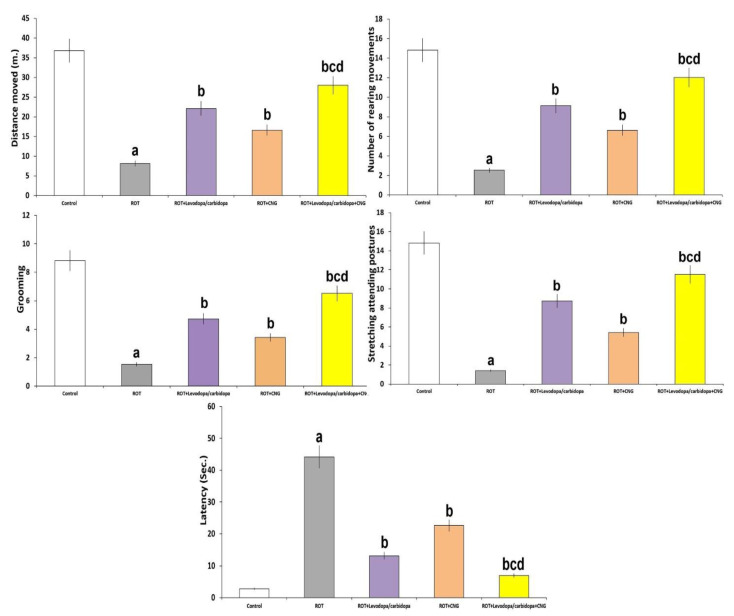
Effect of levodopa/carbidopa with or without canagliflozin on rotenone-induced changes in the open field test (Mean ± SD). ^a^ Significant compared to the control group (*p*-value less than 0.05); ^b^ significant relative to rotenone group (*p*-value less than 0.05); ^c^ significant relative to rotenone + levodopa/carbidopa group (*p*-value less than 0.05); ^d^ significant relative to rotenone + canagliflozin group (*p*-value less than 0.05). ROT (rotenone); CNG (canagliflozin).

The effects of the different treatments on the open field test were demonstrated in Figure 4. Rotenone-treated mice exhibited a significant decline in the distance moved (*p* < 0.001), number of rearing movements (*p* < 0.001), grooming (*p* < 0.01), and stretching postures (*p* < 0.01), while they showed a significant increase in immobile latency (*p* < 0.001) when compared with the control mice. These changes were mitigated with administration of either levodopa/carbidopa or canagliflozin to rotenone-treated mice, with the most favorable results being encountered in mice treated with a combination of levodopa/carbidopa and canagliflozin.

**Figure 5:**
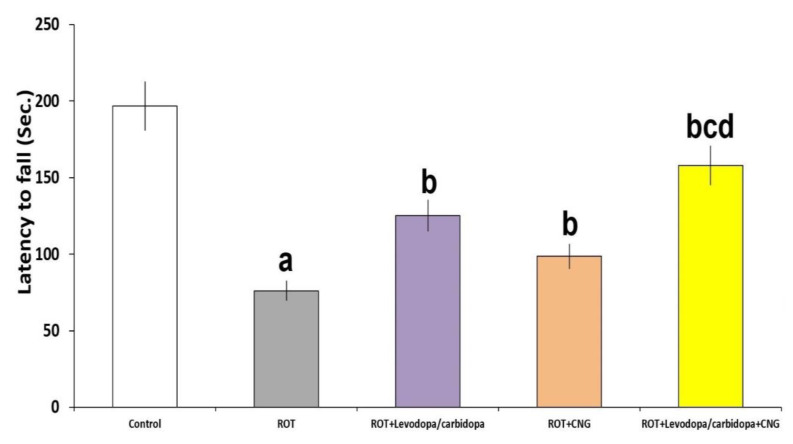
Effect of levodopa/carbidopa with or without canagliflozin on rotenone-induced changes in the rotarod test (Mean ± SD). ^a^ Significant compared to the control group (*p*-value less than 0.05); ^b^ significant relative to rotenone group (*p*-value less than 0.05); ^c^ significant relative to rotenone + levodopa/carbidopa group (*p*-value less than 0.05); ^d^ significant relative to rotenone + canagliflozin group (*p*-value less than 0.05). ROT (rotenone); CNG (canagliflozin).

Figure 5 represents the changes induced by the different treatments in the rotarod test. Rotenone administration was associated with a significant decrease in latency to fall, relative to the control group (*p* < 0.001). Treatment with either levodopa/carbidopa or canagliflozin significantly increased the latency to fall, when compared with the group treated with rotenone alone (*p* < 0.01). This effect was clearly obvious in mice treated with levodopa/carbidopa concomitantly with canagliflozin when compared with mice treated with each of these drugs alone (*p* < 0.01).

### 3.2. Effect of Canagliflozin with or without Levodopa/Carbidopa on the Changes in the Striatal Dopamine Levels Induced by Rotenone Administration

**Figure 6:**
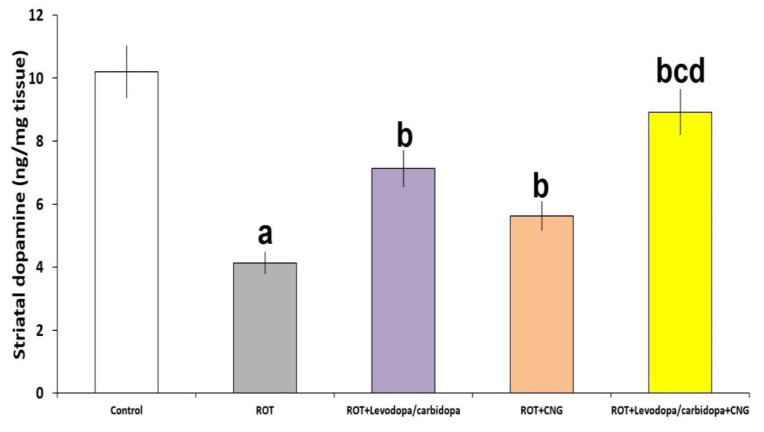
Effect of levodopa/carbidopa with or without canagliflozin on rotenone-induced changes in the striatal dopamine levels (Mean ± SD). ^a^ Significant compared to the control group (*p*-value less than 0.05); ^b^ significant relative to rotenone group (*p*-value less than 0.05); ^c^ significant relative to rotenone + levodopa/carbidopa group (*p*-value less than 0.05); ^d^ significant relative to rotenone + canagliflozin group (*p*-value less than 0.05). ROT (rotenone); CNG (canagliflozin).

Rotenone administration produced a significant decrement in striatal dopamine levels when compared with the control animals (*p* < 0.001). Each of levodopa/carbidopa and canagliflozin administered to rotenone-treated mice significantly elevated the dopamine levels in the striatal tissues when compared with the group treated with rotenone alone (*p* = 0.013 and *p* = 0.021, respectively) but this effect was maximal in the group treated with levodopa/carbidopa concomitantly with canagliflozin when compared with the animal groups treated with each of these drugs alone (*p* = 0.026 vs. levodopa/carbidopa and *p* = 0.008 vs. canagliflozin) (Figure 6).

### 3.3. Effect of Canagliflozin with or without Levodopa/Carbidopa on the Changes in the Levels of Nrf2 and the Redox State of the Striatal Tissues Induced by Rotenone Administration

**Figure 7:**
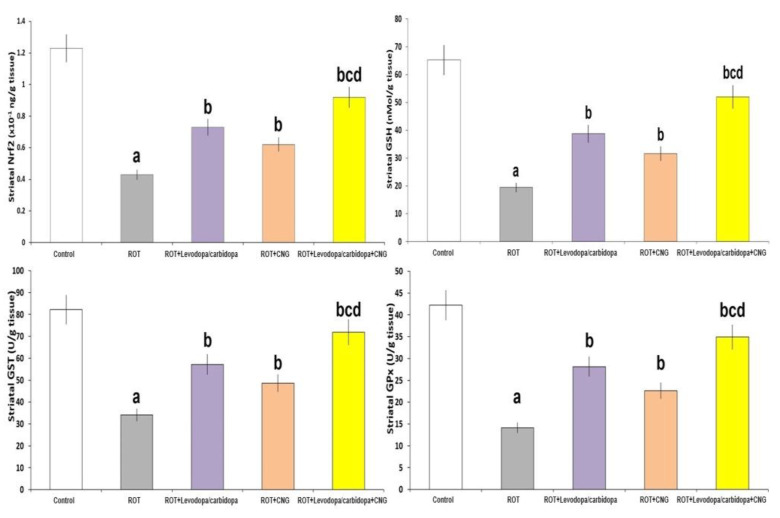
Effect of levodopa/carbidopa with or without canagliflozin on rotenone-induced changes in the levels of Nrf2 and the redox state of the striatal tissues (Mean ± SD). ^a^ Significant compared to the control group (*p*-value less than 0.05); ^b^ significant relative to rotenone group (*p*-value less than 0.05); ^c^ significant relative to rotenone + levodopa/carbidopa group (*p*-value less than 0.05); ^d^ significant relative to rotenone + canagliflozin group (*p*-value less than 0.05). ROT (rotenone); CNG (canagliflozin).

Rotenone administration elicited a significant decrement in the striatal tissue levels of Nrf2 (*p* = 0.003), GSH (*p* < 0.001), and the antioxidant enzymes (*p* < 0.001) relative to the control animals. Treatment of rotenone-injected mice with either levodopa/carbidopa or canagliflozin significantly elevated the striatal tissue levels of Nrf2, GSH, and the antioxidant enzymes when compared with the group treated with rotenone alone. These changes were significant in mice treated with levodopa/carbidopa concomitantly with canagliflozin when compared against the levels of the aforementioned parameters in the animal groups treated with each of these drugs alone (Figure 7).

### 3.4. Effect of Canagliflozin with or without Levodopa/Carbidopa on the Perturbations in the Striatal Tissue Levels of TLR4, TNF-α, IL-1β, and NF-kB (p65) Induced by Rotenone Administration

**Figure 8:**
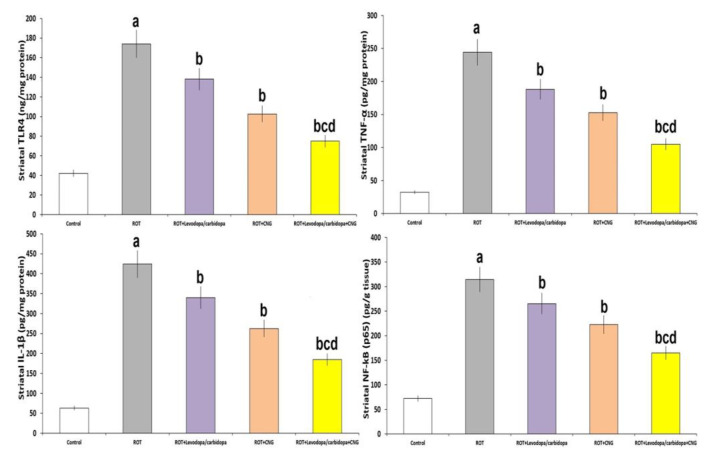
Effect of levodopa/carbidopa with or without canagliflozin on rotenone-induced changes in the levels of toll-like receptor 4 (TLR4), tumor necrosis factor alpha (TNF-α), interleukin 1 beta (IL-1β) and nuclear factor kappa B (NF-kB) p65 in the striatal tissues (Mean ± SD). ^a^ Significant compared to the control group (*p*-value less than 0.05); ^b^ significant relative to rotenone group (*p*-value less than 0.05); ^c^ significant relative to rotenone + levodopa/carbidopa group (*p*-value less than 0.05); ^d^ significant relative to rotenone + canagliflozin group (*p*-value less than 0.05). ROT (rotenone); CNG (canagliflozin).

As depicted in Figure 8, rotenone administration was associated with significantly elevated levels of TLR4, TNF-α, IL-1β, and NF-kB (p65) in the striatal tissues versus the levels of the same parameters in the striatal tissues of the control animals (*p* < 0.001). Animals treated with either levodopa/carbidopa or canagliflozin exhibited significant decline in striatal tissue levels of the aforementioned parameters when compared to animals treated with rotenone alone, but the animal group treated with levodopa/carbidopa concomitantly with canagliflozin had shown the upper hand in decreasing the striatal tissue levels of these parameters over the animal groups treated with each of these drugs alone.

### 3.5. Effect of Canagliflozin with or without Levodopa/Carbidopa on the Perturbations of the Levels of SIRT1 and PPAR-Gamma in the Striatal Tissues Elicited by Rotenone Administration

**Figure 9:**
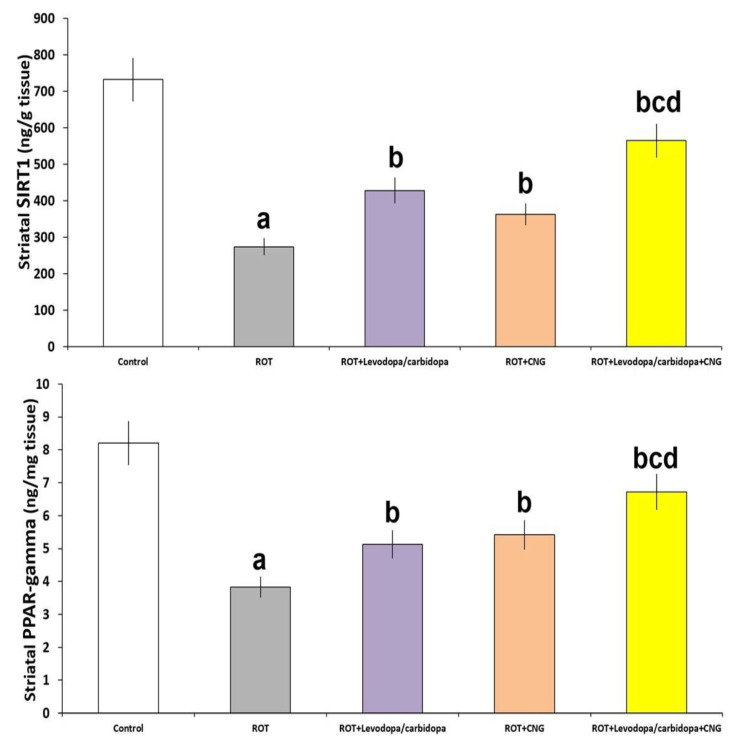
Effect of levodopa/carbidopa with or without canagliflozin on rotenone-induced changes in the striatal tissue levels of sirtuin 1 (SIRT1) and peroxisome proliferator-activated receptor (PPAR)-gamma (Mean ± SD). ^a^ Significant compared to the control group (*p*-value less than 0.05); ^b^ significant relative to rotenone group (*p*-value less than 0.05); ^c^ significant relative to rotenone + levodopa/carbidopa group (*p*-value less than 0.05); ^d^ significant relative to rotenone + canagliflozin group (*p*-value less than 0.05). ROT (rotenone); CNG (canagliflozin).

As demonstrated in Figure 9, the rotenone-induced decrease in SIRT1 levels (*p* < 0.001 vs. control) associated with the significant decline in PPAR-gamma levels (*p* < 0.001 vs. control) in the striatal tissues were ameliorated with the administration of either canagliflozin or levodopa/carbidopa. These admirable effects were more pronounced in the group treated with levodopa/carbidopa concomitantly with canagliflozin when compared with the animal groups treated with each of these drugs alone.

### 3.6. Effect of Canagliflozin with or without Levodopa/Carbidopa on the Changes of the Levels of HMGB1 and RAGE in the Striatal Tissues Elicited by Rotenone Injection

**Figure 10:**
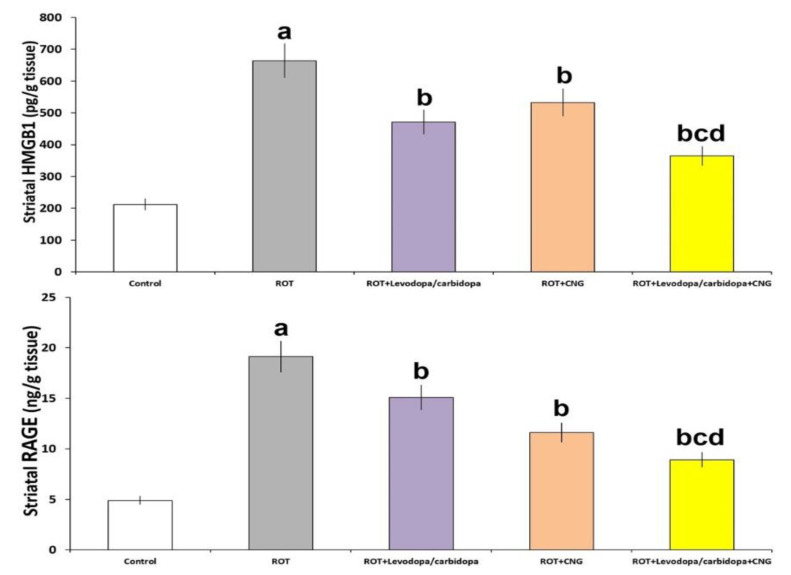
Effect of levodopa/carbidopa with or without canagliflozin on rotenone-induced changes in the striatal tissue levels of HMGB1 and receptors of advanced glycation end products (RAGE) (Mean ± SD). ^a^ Significant compared to the control group (*p*-value less than 0.05); ^b^ significant relative to rotenone group (*p*-value less than 0.05); ^c^ significant relative to rotenone + levodopa/carbidopa group (*p*-value less than 0.05); ^d^ significant relative to rotenone + canagliflozin group (*p*-value less than 0.05). ROT (rotenone); CNG (canagliflozin).

As shown in Figure 10, rotenone injection was accompanied by significantly elevated levels of HMGB1 and RAGE in the striatal tissues when compared against the levels of the same parameters in the striatal tissues of the control animals (*p* = 0.005 and *p* < 0.001 respectively). Animals treated with either levodopa/carbidopa or canagliflozin had significantly lowered striatal tissue levels of HMGB1 and RAGE when compared with mice treated with rotenone alone, but the maximal decline in the striatal tissue levels of these parameters was significantly apparent in the animal group treated with levodopa/carbidopa concomitantly with canagliflozin, relative to the animal groups treated with each of these drugs alone.

### 3.7. Effect of Canagliflozin with or without Levodopa/Carbidopa on the Perturbations in the Levels of Mitochondrial ATP, Mitochondrial Complex-I Activity, and Mitochondrial Transmembrane Potential in the Striatal Tissues Created by Rotenone Administration

**Figure 11:**
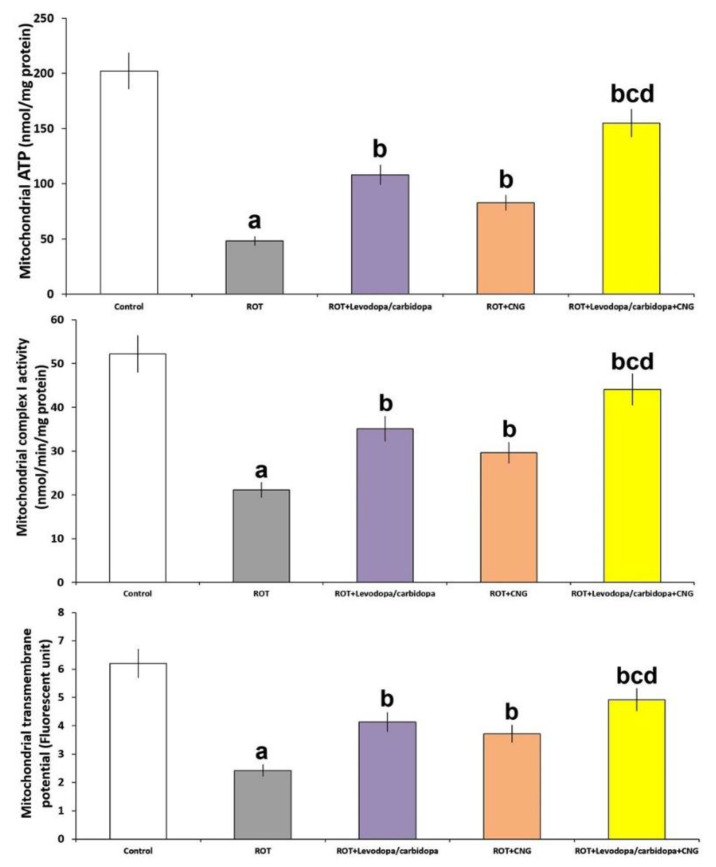
Effect of levodopa/carbidopa with or without canagliflozin on rotenone-induced changes in the mitochondrial functions (Mean ± SD). ^a^ Significant compared to the control group (*p*-value less than 0.05); ^b^ significant relative to rotenone group (*p*-value less than 0.05); ^c^ significant relative to rotenone + levodopa/carbidopa group (*p*-value less than 0.05); ^d^ significant relative to rotenone + canagliflozin group (*p*-value less than 0.05). ROT (rotenone); CNG (canagliflozin).

Figure 11 demonstrates the effect of the different treatments on the mitochondrial functions in the tested animals. Rotenone injection elicited a significant decline in mitochondrial ATP (*p* < 0.001), mitochondrial complex-I activity (*p* < 0.001), and mitochondrial transmembrane potential (*p* = 0.008) in the striatal tissues when compared with the control mice. Levodopa/carbidopa and canagliflozin each exhibited an interesting ability to reverse these changes in rotenone-treated mice with the best results being detected in the animal group treated with levodopa/carbidopa in combination with canagliflozin when compared against the animal groups treated with each of these drugs alone.

### 3.8. Effect of Canagliflozin with or without Levodopa/Carbidopa on the Changes of the Levels of AMPK and mTOR in the Striatal Tissues Elicited by Rotenone Administration

**Figure 12:**
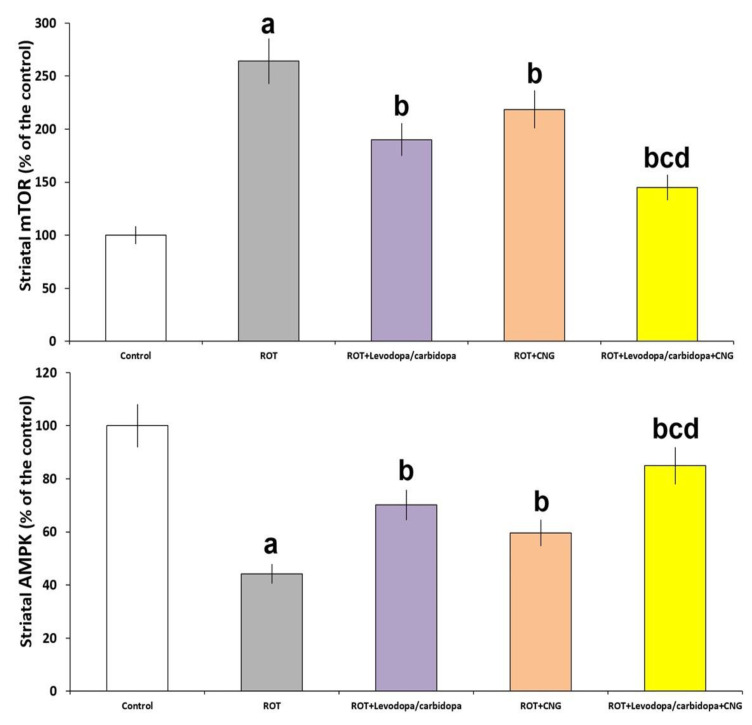
Effect of levodopa/carbidopa with or without canagliflozin on rotenone-induced changes in the striatal tissue levels of AMPK and mTOR (Mean ± SD). ^a^ Significant compared to the control group (*p*-value less than 0.05); ^b^ significant relative to rotenone group (*p*-value less than 0.05); ^c^ significant relative to rotenone + levodopa/carbidopa group (*p*-value less than 0.05); ^d^ significant relative to rotenone + canagliflozin group (*p*-value less than 0.05). ROT (rotenone); CNG (canagliflozin).

Figure 12 demonstrates that rotenone injection was associated with a significant decline in AMPK levels (*p* < 0.001) and associated with significantly increased levels of mTOR (*p* < 0.0001) in the striatal tissues relative to the control group. Treatment with either levodopa/carbidopa or canagliflozin significantly mitigated these changes, with significant elevation in AMPK levels (*p* = 0.011 and 0.02, respectively) and significant decline in mTOR levels (*p* = 0.009 and 0.016, respectively) relative to rotenone-treated mice; but levodopa/carbidopa + canagliflozin combination had the upper hand over the use of each of these agents alone.

### 3.9. Effect of Canagliflozin with or without Levodopa/Carbidopa on the Perturbations in the Autophagy Markers in the Striatal Tissues Induced by Rotenone Administration

**Figure 13:**
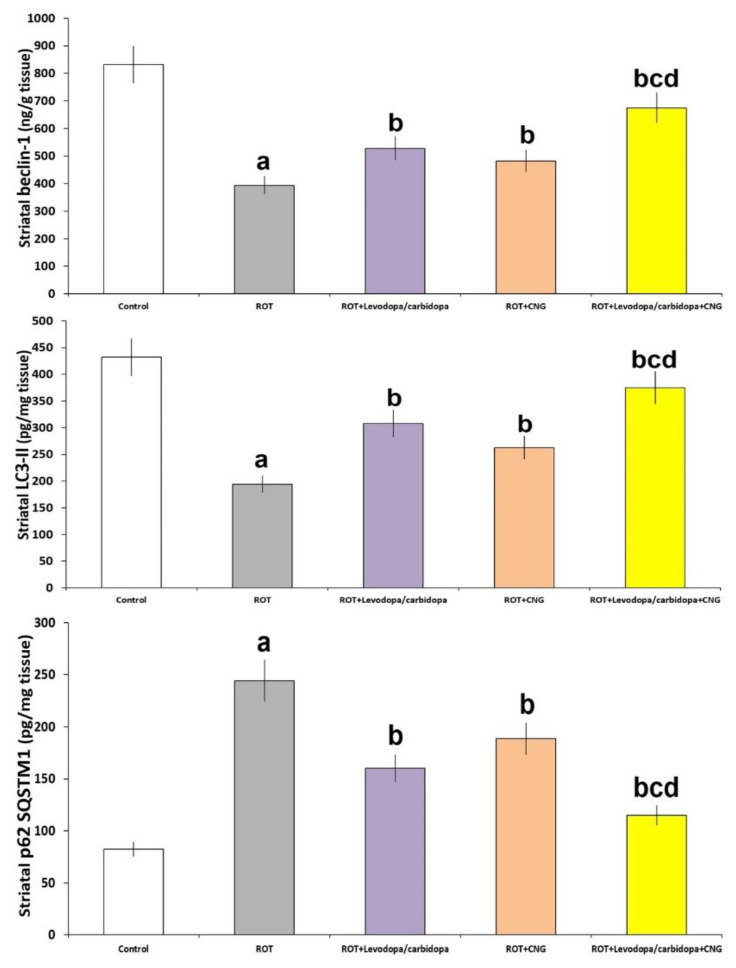
Effect of levodopa/carbidopa with or without canagliflozin on rotenone-induced changes in the autophagy markers in the striatal tissues (Mean ± SD). ^a^ Significant compared to the control group (*p*-value less than 0.05); ^b^ significant relative to rotenone group (*p*-value less than 0.05); ^c^ significant relative to rotenone + levodopa/carbidopa group (*p*-value less than 0.05); ^d^ significant relative to rotenone + canagliflozin group (*p*-value less than 0.05). ROT (rotenone); CNG (canagliflozin).

As depicted in Figure 13, rotenone administration significantly inhibited autophagy, as evidenced by a significant decline in beclin-1 (*p* < 0.001) and LC3-II (*p* < 0.001) levels associated with significant elevation of p62 SQSTM1 levels (*p* < 0.01) in the striatal tissues relative to the control mice. Interestingly, administration of either levodopa/carbidopa or canagliflozin showed an ability to ameliorate these changes in rotenone-treated animals, with the most pronounced results being evidenced in mice treated with levodopa/carbidopa + canagliflozin combination when compared with mice treated with each of these drugs alone.

### 3.10. Effect of Canagliflozin with or without Levodopa/Carbidopa on the Histomorphic Changes in the Brain Tissues Induced by Rotenone Administration

**Figure 14:**
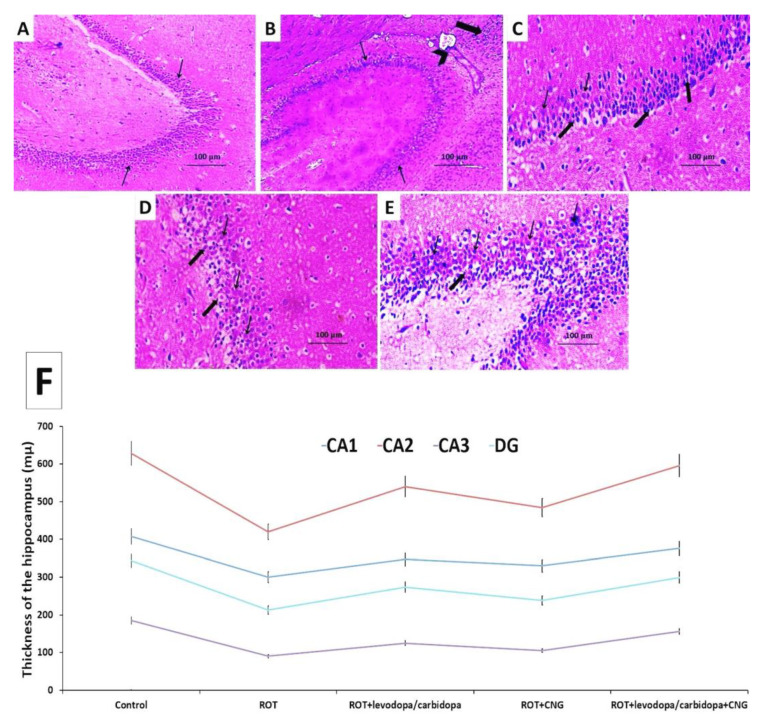
Hematoxylin- and eosin-stained sections from the hippocampus of: (**A**) the control group demonstrating multiple compact layers of pyramidal cells with polygonal cell bodies and vesicular nuclei (thin arrows); (**B**) rotenone-injected group showing significant diminution of the thickness of the pyramidal cell layer, with many cells showing apoptotic changes (thin arrows), diffuse inflammatory cellular infiltration (thick arrow), and marked vascular congestion (arrow head); (**C**) rotenone-injected group treated with levodopa/carbidopa and (**D**) rotenone-injected group treated with canagliflozin exhibiting moderate decline in the number of cells that showed apoptotic changes (thick arrows) with a significantly increased number of normal cells with vesicular nuclei and prominent nucleoli (thin arrows); (**E**) rotenone-injected group treated with levodopa/carbidopa concomitantly with canagliflozin exhibiting marked increase in the number of normal neurons with vesicular nuclei (thin arrows), with scanty dystrophic apoptotic neurons in between (thick arrow); (**F**) the average thickness of the different areas of the hippocampus (Mean ± SD). ROT (rotenone); CNG (canagliflozin); DG (dentate gyrus).

The hippocampus of the group that received rotenone alone exhibited significant diminution of the thickness of the pyramidal cell layer, with many cells showing apoptotic changes, diffuse inflammatory cellular infiltration, and marked vascular congestion (Figure 14A,B) associated with a significant decrease in the thickness of the different areas of the hippocampus relative to the control group (Figure 14F). Administration of either levodopa/carbidopa or canagliflozin to rotenone-injected animals resulted in a moderate decline in the number of the cells that showed apoptotic changes, with a significantly increased number of normal cells (Figure 14C and Figure 14D, respectively) associated with a significant increase in the thickness of the different areas of the hippocampus when compared with animals that received rotenone alone. The rotenone-injected group treated with levodopa/carbidopa concomitantly with canagliflozin exhibited a significant increase in the number of normal neurons, with scanty dystrophic apoptotic neurons in between (Figure 14E) associated with significant restoration of the thickness of the different areas of the hippocampus, to approximate the control values (Figure 14F).

**Figure 15:**
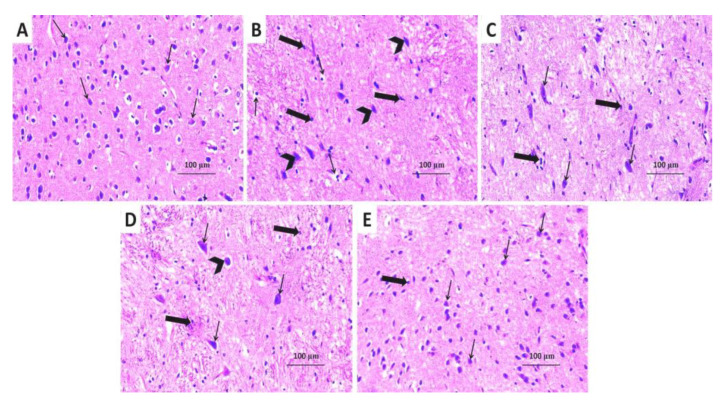
Hematoxylin- and eosin-stained sections from the substantia nigra of (**A**) the control group demonstrating the dopaminergic neurons with vesicular nuclei and basophilic cytoplasm (thin arrows); (**B**) rotenone-injected group showing massive neurodegeneration. The neurons appear small and shrunken (thick arrows) with many neurons showing irregular outlines, cytoplasmic shrinkage, and pyknotic darkly stained nuclei (arrow heads) with perineuronal vacuolations (thin arrows); (**C**) rotenone-injected group treated with levodopa/carbidopa; and (**D**) rotenone-injected group treated with canagliflozin exhibiting a moderate decline in the number of the degenerated shrunken cells (thick arrows) and the nuclei showing pyknotic changes (arrow head) with significantly increased number of the normal dopaminergic neurons with vesicular nuclei (thin arrows); (**E**) rotenone-injected group treated with levodopa/carbidopa concomitantly with canagliflozin exhibiting significant increase in the number of the normal dopaminergic neurons with vesicular nuclei (thin arrows) associated with scanty small shrunken neurons in-between (thick arrow).

When compared with the control group, the substantia nigra of rotenone-treated mice exhibited significant decrement in the number and size of the neuronal cells which show degenerative changes manifested as irregular outlines, shrinkage of the cytoplasm, chromatin condensation in the pyknotic nuclei, and perineuronal vacuolation (Figure 15A,B). Treatment of rotenone-injected mice with either levodopa/carbidopa or canagliflozin induced evident improvement in the histomorphic features of the neuronal cells, with apparently normal cellular outlines with few cytoplasmic inclusions and scattered areas of perineuronal vacuolation (Figure 15C,D); with the maximal degree of improvement being detected in mice concomitantly treated with levodopa/carbidopa and canagliflozin relative to the animal groups treated with each of these drugs alone (Figure 15E).

### 3.11. Effect of Administration of Canagliflozin with or without Levodopa/Carbidopa on TH Immunostaining in the Substantia Nigra of Rotenone-Treated Mice

**Figure 16:**
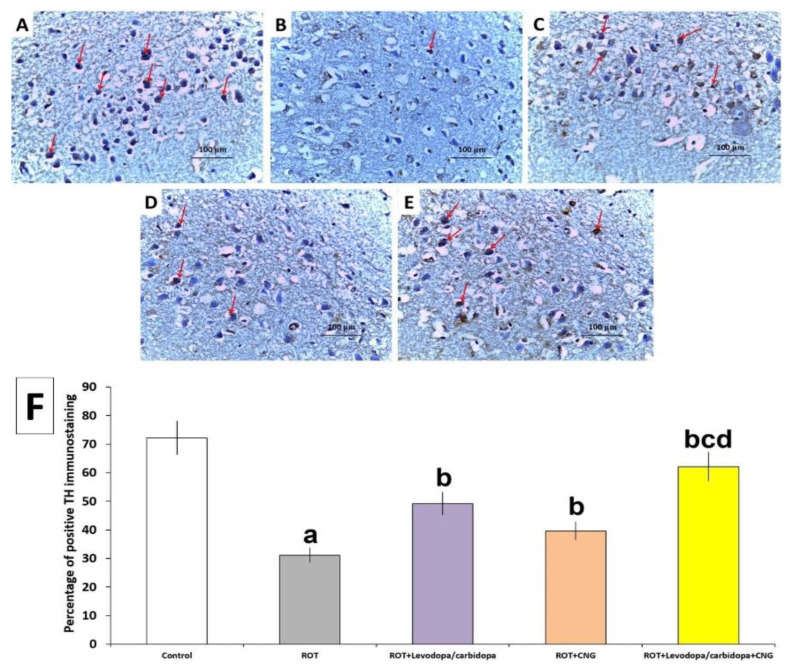
The immunohistochemical staining of tyrosine hydroxylase (TH) (×40) in the tissue sections of the substantia nigra of: (**A**) the control group exhibiting strong positive TH immunostaining; (**B**) rotenone-injected group showing minimal positive TH immunostaining; (**C**) rotenone-injected group treated with levodopa/carbidopa; and (**D**) rotenone-injected group treated with canagliflozin demonstrating moderately positive TH immunostaining; (**E**) rotenone-injected group treated with levodopa/carbidopa concomitantly with canagliflozin revealing strong positive TH immunostaining; (**F**) the percentage of positive immunostaining of TH in the substantia nigra (Mean ± SD). ^a^ Significant compared to the control group (*p*-value less than 0.05); ^b^ significant relative to rotenone group (*p*-value less than 0.05); ^c^ significant relative to rotenone + levodopa/carbidopa group (*p*-value less than 0.05); ^d^ significant relative to rotenone + canagliflozin group (*p*-value less than 0.05). ROT (rotenone); CNG (canagliflozin).

Rotenone administration significantly decreased TH immunoexpression when compared with the control group (Figure 16A,B,F). Treatment of rotenone-injected mice with either levodopa/carbidopa or canagliflozin significantly increased the degree of TH immunoexpression relative to mice treated with rotenone alone (Figure 16C,D,F). Mice concomitantly treated with levodopa/carbidopa and canagliflozin exhibited a significant increase in TH immunoexpression when compared with the animals treated with each of these drugs alone (Figure 16E,F).

## 4. Discussion

In the present study, we utilized the chronic rotenone model to induce PD according to previous studies that led to neuronal depletion of dopamine [39]. It is worth noting that dopamine depletion is the cornerstone of PD pathogenesis [40]. Therefore, it was chosen as an animal model of PD to evaluate the potential therapeutic effect of canagliflozin alone or in combination with L-dopa and carbidopa, which is the main goal of our study.

The present study demonstrated that the rotenone-induced PD group was manifested by statistically significant deficits in behavior as compared to the control group. These results agree with previous studies [41] in which the behavioral impairment referred to the loss of dopaminergic neurons that correlates with a significant decrement in the striatal dopamine levels versus the control group. Tyrosine hydroxylase is the rate-limiting enzyme in dopamine synthesis [42]. Hence, rotenone significantly decreased the tyrosine hydroxylase level demonstrated in immunohistochemical staining of the tissue and is compatible with the neuronal cell depletion and striatal tissue degeneration in histopathological examination that correlate with previous studies [43]. We found that administration of canagliflozin to the rotenone group led to a significant restoration of dopamine level and tyrosine hydroxylase level that was reflected in the amelioration of behavior deficits, as well as preserving neuronal tissue integrity. The ameliorative effect of canagliflozin can be explained as it has a neuroprotective effect. In addition, SGLT2 inhibitors are antidiabetic drugs, and there have been emerging shreds of evidence connecting PD and diabetes mellitus recently [44].

In our study, we supposed that the pathogenesis of rotenone-induced PD depends on mitochondrial dysfunction revealed by a significant decline in mitochondrial transmembrane potential, mitochondrial complex-I activity, and mitochondrial ATP. These results are similar to those of previous studies [45], which explain that mitochondrial dysfunction leads to oxidative stress induction and antioxidant activity feebleness; as proved in our study via a significant decrement in the striatal tissue levels of GSH, the antioxidant enzymes relative to the control group. Hence, rotenone also decreased tissue Nrf2 level, which regulates the expression of antioxidant-related genes and resistance to reactive oxygen species [46]. Our findings of the redox state of tissues are in agreement with previous studies [47]. Canagliflozin has antioxidant properties and can preserve the activity of antioxidant enzymes [48], leading to amelioration of mitochondrial dysfunction; this is observed in our study through a significant restoration of the tissue redox, and these results agree with previous studies [49,50]. Ma et al. [51] showed that canagliflozin can modulate Nrf2 signaling in tissue, which correlates with our finding. Activation of Nrf2 has an important role in the amelioration of oxidative stress and neuroinflammation in PD [52].

Neuroinflammation has a pivotal role in PD pathogenesis [53]. The activated microglia release inflammatory cytokines such as tumor necrosis factor-α (TNF-α) and interleukin-1β, leading to degeneration of dopaminergic neurons [54]. Rotenone-induced neuroinflammation in PD pathophysiology is associated with the upregulation of HMGB1 and RAGE, with subsequent increased proinflammatory cytokines [55]. HMGB1 is released from necrotic or apoptotic cells in response to TNF-α, depending on the NF-κB pathway. HMGB1 itself can induce more inflammatory cytokine release via interaction with TLRs and RAGE. RAGE and TLRs stimulate the immune system and cellular migration into inflamed tissue [56]. Stimulation of TLRs leads to more activation of NF-kB transcription [57]. Our study supports this point, which was demonstrated by a significant elevation of HMGB1, RAGE, TLR4, TNF-α, and NF-kB levels in the striatal tissues versus the control group. Canagliflozin has anti-inflammatory activity via a decrease in inflammatory cell activation, inflammatory cytokine release [58], and downregulation of HMGB1, RAGE, TLR4, and NF-kB signaling [59]. Herein, we observed a significant decrease in the level of HMGB1, RAGE, TLR4, TNF-α, and NF-kB levels in the group treated with canagliflozin versus rotenone in the striatal tissues.

Canagliflozin’s ability to refine neuroinflammation in our study was also confirmed by a significant increase of SIRT1, PPAR-gamma, and AMPK levels versus the rotenone group, which is similar to previous studies [60]. SIRT1 is a protein with histone deacetylase activity leading to inhibition of the inflammatory transcription such as NF-κB P65 subunit, and subsequent decrease of its activity [61]. SIRT1 also can indirectly suppress the NF-κB pathway via regulating the AMPK and PPAR expression. SIRT1 activates AMPK, which is a vital inhibitor of NF-κB [62]. Additionally, canagliflozin can increase the activity of AMPK and is associated with suppression of mitochondrial complex 1 inhibition and diminished inflammatory cytokine release [58]. SIRT1 overexpression is associated with enhancing the interaction between P65 and PPARα with a subsequent decrease in the NF-κB activation, thus inhibiting the transcription of the inflammatory cytokines [63]. Moreover, PPARγ acts as an anti-inflammatory factor via modulating the immune inflammatory response and negative regulation of inflammatory cells such as macrophages and monocytes, with subsequent suppression of pro-inflammatory cytokines production, such as TNF-α and IL-6 [64]. Overexpressed PPAR-gamma can protect dopaminergic neurons from degeneration and regulate glial cell activation in PD model [65]

The pathophysiology of rotenone-induced PD is not fully described via the oxidative neuroinflammatory pathway, despite it being an important initiating pathway. Autophagy also has a pivotal role in the pathophysiology of PD [34]. Autophagy is a cytoprotective mechanism which is a result of cellular stress exposure to preserve cellular homeostasis [66]. It also can deplete reactive oxygen species (ROS) and regulate redox signaling [67]. Several studies have linked impairment of the autophagy–lysosome pathway in PD animal models with postmortem striatal tissue of PD patients [68]. Rotenone can inhibit autophagy induction by enhancement of mTOR in striatal tissue and, as a result, Beclin-1 expression and LC3-II levels decrease [69,70]. Rotenone can also impair the autophagic flux represented by increased SQSTM1/p62 level [71]. This autophagy impairment yields upregulation of NF-kB transcription and an increase in proinflammatory cytokines [72]. Our study is in harmony with this research point, which was proved by a significant decrease in LC3-II and Beclin-1 levels, and a significant increase in mTOR and SQSTM1/P62 levels as compared to the control group. Interestingly, we observed that treatment with canagliflozin led to a decrease of mTOR and SQSTM1/P62 levels induced by rotenone administration and an increase in LC3-II and Beclin-1 levels, which is a clear indication of canagliflozin’s ability to induce the autophagy process, and these findings are in harmony with previous studies [73,74] that reported that SGLT2 inhibitors such as canagliflozin can stimulate autophagy and induce the autophagy flux, leading to amelioration of neuroinflammation. Its stimulatory effect on autophagy cascade was initially demonstrated in previous studies on diabetic animals [75] where the authors have explained this effect via indirect stimulation of autophagic flux through fasting-like conditions, leading to transcription of different genes that regulate the autophagy pathway [76]. Recently, several studies have shown a relationship between the protective role of canagliflozin and the activation of autophagy. The anti-inflammatory properties of canagliflozin can be attributed to its promoting effect on autophagy via mTOR suppression and AMPK activation [77]. AMPK upregulates autophagy through negative regulation of mTOR and phosphorylation of ULK1 with subsequent initiation of the autophagy cascade and formation of autophagic vacuoles [78].

Levodopa is considered the most efficient therapy for PD [3]. However, long-term therapy with levodopa is usually associated with adverse effects such as dyskinesia, which is defined as abnormal voluntary movements that can be explained by dopaminergic neuron overstimulation [55]. Therefore, more research is being conducted to find a new adjuvant therapy that improves PD manifestations with less or no dyskinesia.

Fortunately, in our study, the use of canagliflozin concomitantly with levodopa/carbidopa treatment enhanced behavioral deficit significantly versus the levodopa/carbidopa treated group. Herein, this difference can be attributed to a significant increase in striatal levels of dopamine and tyrosine hydroxylase enzyme versus the levodopa/carbidopa group. As mentioned above, canagliflozin can ameliorate the pathology of PD in striatal tissue via restoration of the redox state towards near normal, and inhibition of neuroinflammation and autophagy stimulation. Therefore, the combination of L-dopa and canagliflozin can significantly induce more reduction in oxidative stress and neuroinflammation and increase in autophagy activity versus the levodopa/carbidopa group. Interestingly, the long-term use of levodopa/carbidopa was reported to elevate blood glucose levels and may contribute to the high prevalence of insulin resistance observed in patients with PD [79]. In addition, normal glycemic state was reported to have a direct impact on the intestinal absorption and utilization of levodopa [80]. This highlights the possible role of canagliflozin in improvement of the efficacy and minimization of the adverse effects of levodopa/carbidopa.

## 5. Conclusions

In view of the findings of the current study, canagliflozin demonstrated promising neuroprotective effects and may boost the therapeutic effect of levodopa/carbidopa in a rotenone-induced PD model, depending on its antioxidant and anti-inflammatory effects in addition to its ability to restore the normal autophagy state. Our ongoing studies will examine whether this combination could also limit dyskinesia induced by levodopa beyond amelioration of the pathophysiology of PD. Also, further experiments are required to explore and verify the precise molecular mechanisms by which canagliflozin with or without levodopa/carbidopa may combat rotenone-induced Parkinson’s disease, including Western blotting and mRNA measurements of the target proteins in addition to experiments on knock-out mice. In addition, assessment of the markers for the mitochondrial mass content such as mtDNA levels or protein levels for mitochondrial mass is vitally needed to highlight the role of mitophagy in the pathogenesis of PD. Furthermore, the observations of the present study should be verified in further human clinical trials.

## Data Availability

The data are available from the corresponding author upon reasonable request.
